# Elevated Type 1 Metabotropic Glutamate Receptor Availability in a Mouse Model of Huntington’s Disease: a Longitudinal PET Study

**DOI:** 10.1007/s12035-019-01866-5

**Published:** 2020-01-08

**Authors:** Daniele Bertoglio, Jeroen Verhaeghe, Špela Korat, Alan Miranda, Klaudia Cybulska, Leonie Wyffels, Sigrid Stroobants, Ladislav Mrzljak, Celia Dominguez, Mette Skinbjerg, Longbin Liu, Ignacio Munoz-Sanjuan, Steven Staelens

**Affiliations:** 1 Molecular Imaging Center Antwerp (MICA), University of Antwerp, Wilrijk, Belgium; 2 Department of Nuclear Medicine, Antwerp University Hospital, Edegem, Belgium; 3 CHDI Management/CHDI Foundation, Los Angeles, CA USA

**Keywords:** [^11^C]ITDM, Glutamate, mGluR1, Brain imaging, Receptor

## Abstract

Impairment of group I metabotropic glutamate receptors (mGluRs) results in altered glutamate signalling, which is associated with several neurological disorders including Huntington’s Disease (HD), an autosomal neurodegenerative disease. In this study, we assessed in vivo pathological changes in mGluR1 availability in the Q175DN mouse model of HD using longitudinal positron emission tomography (PET) imaging with the radioligand [^11^C]ITDM. Ninety-minute dynamic PET imaging scans were performed in 22 heterozygous (HET) Q175DN mice and 22 wild-type (WT) littermates longitudinally at 6, 12, and 16 months of age. Analyses of regional volume of distribution with an image-derived input function (*V*_T (IDIF)_) and voxel-wise parametric *V*_T (IDIF)_ maps were performed to assess differences between genotypes. *Post-mortem* evaluation at 16 months was done to support in vivo findings. [^11^C]ITDM *V*_T (IDIF)_ quantification revealed higher mGluR1 availability in the brain of HET mice compared to WT littermates (e.g. cerebellum: + 15.0%, + 17.9%, and + 17.6% at 6, 12, and 16 months, respectively; *p* < 0.001). In addition, an age-related decline in [^11^C]ITDM binding independent of genotype was observed between 6 and 12 months. Voxel-wise analysis of parametric maps and *post-mortem* quantifications confirmed the elevated mGluR1 availability in HET mice compared to WT littermates. In conclusion, in vivo measurement of mGluR1 availability using longitudinal [^11^C]ITDM PET imaging demonstrated higher [^11^C]ITDM binding in extra-striatal brain regions during the course of disease in the Q175DN mouse model.

**Electronic supplementary material:**

The online version of this article (10.1007/s12035-019-01866-5) contains supplementary material, which is available to authorized users.

## Introduction

Glutamate is the primary excitatory neurotransmitter in the central nervous system and exerts its action through both ionotropic and metabotropic receptors. Group I metabotropic glutamate receptors (mGluRs) are post-synaptic G protein-coupled receptors and include mGluR type 1 (mGluR1) and type 5 (mGluR5) [1]. These receptors mainly promote intracellular Ca^2+^ release and protein kinase C activation [2]. Impairment in their functionality can cause altered glutamate signalling and consequently excitotoxicity, a reason why both mGluR1 and mGluR5 have been associated with several neurological disorders, including Huntington’s Disease (HD) [3, 4].

HD is an inherited autosomal dominant neurodegenerative disorder caused by an expanded polyglutamine (CAG) repeat in the exon 1 of the *HTT* gene [5]. This mutated gene translates to mutant huntingtin (mHTT), which is the causative agent of the disease. As a consequence of mHTT accumulation, neuronal dysfunction and death occurs, leading to progressive motor, psychiatric, and cognitive impairments in individuals with HD [6, 7].

Of note, mGluR1 and mGluR5 display distinct cerebral expression patterns: mGluR5 is highly expressed in striatum, hippocampus, and cortex, whereas mGluR1 has a primarily thalamic and cerebellar distribution, with low levels in the other brain regions [8, 9].

Thus, likely due to the striatal and cortical distribution, previous studies mainly focused on mGluR5 and described altered receptor density in both HD mice [10] and human *post-mortem* tissue [11], although an understanding of the underlying mechanism is still a topic of debate. Knockout and pharmacological antagonism of mGluR5 have been shown to reduce formation of mHTT aggregates [12, 13], whilst mGluR5 positive allosteric modulation improved synaptic plasticity [14, 15]. Additionally, we recently characterized the longitudinal changes of mGluR5 density occurring during disease progression in the Q175 mouse model of HD [16] by means of positron emission tomography (PET) and found that mGluR5 levels were decreased in HD mice [17].

On the contrary, mGluR1 has received limited attention, and little is known on possible changes in mGluR1 availability during the progression of HD. However, the extra-striatal distribution of mGluR1 is exactly in regions implicated in movement disorders, such as the cerebellum and thalamus, and it is thus of high relevance, among others, to HD [3]. The aim of this study was to investigate whether changes in mGluR1 availability occur during disease progression in the same mouse model of HD we used to quantify mGluR5 [17] in order to provide a first evidence before clinical investigation. We performed longitudinal non-invasive PET imaging of mGluR1 at 6, 12, and 16 months of age using the selective radioligand [^11^C]ITDM (N-[4-[6-(isopropylamino)-pyrimidin-4-yl]-1,3-thiazol-2-yl]-N-methyl-4-[^11^C]-methylbenzamide) [18], for which we recently validated the pharmacokinetic methodology to perform accurate quantification of mGluR1 availability in the mouse brain [19]. By using the same Q175 mouse model, these findings will offer new insights into the characterization of both mGluR1 and mGluR5 during HD progression.

## Materials and Methods

### Animals

Thirty-seven 6 months old male heterozygous (HET) Q175DN mice (C57BL/6J background) and 37 age-matched wild-type (WT) littermates were obtained from the Jackson Laboratories (Bar Harbour, Maine, USA). Since C57BL/6J mice present sporadic congenital portosystemic shunt [20], animals were screened before inclusion in the study to avoid this variable as confounding factor. This mouse model of HD exhibits motor, cognitive, molecular, and electrophysiological abnormalities, including in vivo decrease in several striatal markers and HD hallmarks similarly to patients with HD [16, 17, 21–24]. Only HET mice were included in the study to better resemble the clinical condition as homozygousity is rare in patients with HD.

Twenty-two mice per genotype were allocated to the longitudinal study, while the remaining ones (*n* = 15/genotype) were used for radiometabolite measurements. Animals were single-housed in individually ventilated cages with temperature- and humidity-controlled environment on a 12 h light/dark cycle with access to food and water ad libitum. Animals were given at least 1 week to habituate to the facility before the start of the procedures.

### Radiotracer Synthesis

[^11^C]ITDM synthesis was performed on an automated synthesis module (Carbosynthon I, Comecer, The Netherlands) optimizing the reported procedure [18] to our system as recently described [19]. Average molar activity was 90.1 ± 27.6 GBq/μmol with radiochemical purity greater than 99%. [^3^H]ITDM was synthetized by Pharmaron, UK, with a molar radioactivity of 2.66 GBq/μmol and purity above 99.9%.

### [^11^C]ITDM Dynamic PET Imaging

#### Image Acquisition

Dynamic microPET/computed tomography (CT) imaging was performed on two Siemens Inveon PET/CT scanners (Siemens Preclinical Solution, Knoxville, USA). Following isoflurane anaesthesia (induction 5%, maintenance 1.5–2% supplemented in oxygen) (Forene, Belgium), animals were catheterized in the tail vein for intravenous (i.v.) bolus injection of the tracer and were placed on the scanner bed. The whole body was in the PET scanner’s field of view to allow the extraction of the image-derived input function (IDIF) from the lumen of the left ventricle of the heart as previously described and validated [19, 25]. A monitoring acquisition module (Minerve, France) was used to constantly monitor the respiration of the animal during the entire preparation and scanning period. The core body temperature of the animals was maintained stable at 37 ± 1 °C using a heating pad during the entire preparation phase and a feedback-controlled warm air flow (Minerve, France) during the scanning period.

[^11^C]ITDM image acquisition was performed as previously validated [19]. At the onset of the 90 min dynamic microPET scan, animals were injected with a bolus of [^11^C]ITDM over a 12 s interval (1 ml/min) using an automated pump (Pump 11 Elite, Harvard Apparatus, USA). Activity was injected in a trace dose, keeping the cold mass similar across time points, and below a maximum limit (2.0 μg/kg). PET scans were acquired in list-mode format and followed by a 10 min 80 kV/500 μA CT scan for attenuation correction and co-registration.

Animals (*n* = 44) were imaged longitudinally at 6, 12, and 16 months of age. A total of 2 WT and 4 HET Q175DN mice died during longitudinal imaging (in part due to recovery related to anaesthesia). Details regarding body weight, molar radioactivity, injected radioactivity and mass, and number of animals for each time point are provided in Supplementary Table 1.

### Metabolite Analysis

In order to assess possible genotypic difference in peripheral metabolism, a population-based metabolite analysis was performed in a cohort of WT (*n* = 15) and HET (*n* = 15) mice at 0, 5, 15, and 30 min post injection (p.i.). The procedure was as previously described [19]. Briefly, mice were injected with the radioligand via the lateral tail vein, and blood was collected via cardiac puncture. Next, samples were centrifuged at 2377 × rcf for 5  min, and both plasma and residual fractions were counted in a gamma counter (Wizard^2^, PerkinElmer). Following addition of equal amounts of ice-cold acetonitrile to the plasma samples and centrifugation at 2377 × rcf for 5 min, supernatant was separated from the precipitate, and both fractions were counted in the gamma counter to calculate the plasma extraction efficiency (WT: 96.4 ± 2.3%; HET: 97.3 ± 0.8%). Finally, 100 μl of supernatant were loaded onto a preconditioned reverse-phase (RP)-HPLC system (Kinetex, 150 × 4.6 mm, 5 μm HPLC column + Phenomenex security guard precolumn), fractions were collected, and radioactivity was measured in the gamma counter to determine the unchanged fraction. Blood spiked in vitro with 37 kBq of radiotracer indicated no degradation occurred during the workup (unchanged radioligand = 99.3 ± 0.4%).

### Image Processing and Data Analysis

Acquired 90 min PET data were histogrammed and reconstructed into 39 frames of increasing length (12 × 10 s, 3 × 20 s, 3 × 30 s, 3 × 60 s, 3 × 150 s, and 15 × 300 s). Images were reconstructed using a list-mode iterative reconstruction with proprietary spatially variant resolution modelling with 8 iterations and 16 subsets of the 3D ordered subset expectation maximization (OSEM 3D) algorithm. Normalization, dead time, and CT-based attenuation corrections were applied. PET image frames were reconstructed on a 128 × 128 × 159 grid with 0.776 × 0.776 × 0.776 mm^3^ voxels. Processing and analysis of the PET images was performed using PMOD 3.6 software (Pmod Technologies, Zurich, Switzerland).

Spatial normalization of the PET images was achieved by normalization of the images to a [^11^C]ITDM PET template as previously described [19, 24]. Time activity curves (TACs) were extracted from the images using a volume of interest brain atlas adapted from the Waxholm one [26]. mGluR1 is primarily expressed in cerebellum and thalamus. Nonetheless, it can be measured at lower levels also in other brain regions, so we considered striatum, motor cortex, and hippocampus given their relevance to HD. Finally, pons was included in order to assess the application of reference region-based analysis.

Kinetic modelling was performed by fitting the TACs using a two-tissue compartment model (2TCM) with a blood volume fraction (*V*_B_) fixed at 3.6% [27] and using the Logan model [28] with the start of the linear phase (t*) calculated according to the maximum error criterion (10%; t* ranging 12.5–30 min depending on the brain region). The total volume of distribution based on image-derived input function (*V*_T (IDIF)_) was calculated as a non-invasive surrogate of the *V*_T_ given the high linear correlation when compared to the invasive approach (r = 0.977, r^2^ = 0.954, *p* < 0.0001) as we recently validated [19].

The IDIF was obtained as recently described [19]. Briefly, using the CT image for anatomical information, a sphere (3.5 mm in diameter) was generated with centre in the lumen of the left ventricle of the heart. Next, by selecting the early PET frame exhibiting maximal activity, a threshold set to 50% of max was applied in order to obtain a stable volume across subjects and standardize the procedure. The volume was then used to extract the activity directly from the PET image. The IDIF measured for each genotype and time point are shown in Supplementary Fig. 1. No radiometabolite correction was applied to the IDIF as no difference in metabolism between genotypes was measured (Supplementary Fig. 2).

Also, since an excellent agreement exists between values obtained with 2TCM and Logan plot (r = 0.993, r^2^ = 0.986, *p* < 0.0001) [19], only *V*_T (IDIF)_ values determined with Logan are reported. Finally, to investigate the consequence of applying a reference region-based analysis on mGluR1 quantification, we estimated the binding potential (*BP*_ND_) using pons as reference region as it was previously reported [29]. In addition to the distribution volume ratio minus 1 (*DVR*-1) [30], calculated as the ratio of *V*_T (IDIF)_ of a target region over the *V*_T (IDIF)_ in the reference region minus 1, we used the simplified reference tissue model 2 (SRTM2) [31]. Given the agreement between the two approaches (r = 0.992, r^2^ = 0.984, *p* < 0.0001), only *BP*_ND_ values based on SRTM2 are reported. The simplified reference tissue model (SRTM) [32] was not included as it failed to fit the data of few subjects. For this reason, the *k*_2_’ values for SRTM2 were based on the average value of each group across different regions.

Parametric *V*_T (IDIF)_ maps were generated in PXMOD (Pmod Technologies, Zurich, Switzerland) through voxel-wise Logan graphical analysis using the IDIF as input function with the t* calculated according to the maximum error criterion (10%). The averages for each genotype were overlaid to a 3D mouse brain template for anatomical reference. Finally, PET images were smoothed using an isotropic Gaussian filter (FWHM = 0.5 mm), and a voxel-wise analysis with statistical parametric mapping (SPM) was performed using SPM12 (Wellcome Department of Imaging Neuroscience, London, UK). For voxel-wise analysis, data obtained from WT and HET Q175DN mice at each time point were compared, considering both contrasts (WT > HET and HET > WT). Statistical T-maps were calculated for a peak voxel threshold of *p* = 0.05 (uncorrected) and cluster threshold of 100 voxels (0.8 mm^3^). Only significant clusters with *p* < 0.05 were considered and reported.

### Brain Tissue Collection

One week following the last PET scan, 16-month old animals (WT: *n* = 20; HET: *n* = 18) were euthanized by decapitation while under anaesthesia, and brains were snap-frozen in 2-metylbuthane at − 35 °C for 2 min and further preserved at − 80 °C until use. Sagittal sections (20 μm of thickness) were collected serially starting at 0.96 mm lateral according to Paxinos and Franklin [33] in triplicate on Superfrost Plus slides (Thermo Fisher Scientific, USA), using a cryostat (Leica, Germany).

### In Vitro [^3^H]ITDM Autoradiography

Autoradiography was performed as recently validated [19]. Briefly, sections were pre-incubated for 20 min with binding buffer (50 mM Tris-HCl buffer, pH 7.4, containing 2 mM CaCl_2_ and 1.2 mM MgCl_2_). Next, 1 h incubation with total binding solution (TB, 0.5 nM of [^3^H]ITDM) or nonspecific-binding solution (NB, 0.5 nM of [^3^H]ITDM +1 μM of cold ITDM) at room temperature. Sections were washed in 50 mM Tris-HCl buffer on ice, followed by distilled water, and dried for 2 h at room temperature. Sections were exposed on imaging plates (BAS-TR2025, Fujifilm, Japan) for 90 h and imaged with a phosphor imager (Fuji FLA-700 image reader). The measured grey intensity was transformed into radioactivity based on intensity values obtained using tritium standards (American Radiolabeled Chemicals Inc., USA). Given the known decay-corrected molar activity of [^3^H]ITDM and tritium standards, the calculated radioactivity was transformed into picomoles.

Regional quantification was performed using ImageJ software (National Institute of Health, USA). Binding was measured in the same regions of interest manually drawn on each section as measured on the PET images. Regional specific binding was measured in triplicate (3 slices) for each region. The average of each animal was used for statistical analysis.

### mGluR1 Immunohistochemistry

In vitro mGluR1 levels were also visualized by immunohistochemistry. First, sections were air dried for 5 min and incubated with 4% paraformaldehyde (PFA) for 15 min as tissue postfixation. Following rinsing with phosphate-buffered saline (PBS, pH 7.4), nonspecific binding sites were blocked using 5% normal goat serum (NGS) and 0.3% Triton X-100 in PBS for 1 h. Next, sections were incubated with the monoclonal primary antibody anti-mGluR1 (rabbit; 1:200; #12551, Cell Signalling Technologies) in antibody diluent containing 1% bovine serum albumin (BSA) and 0.3% Triton X-100 in PBS overnight at room temperature. The next day, sections were rinsed with PBS prior to a 1 h incubation with the secondary antibody (goat anti-rabbit conjugated with horseradish peroxidase; 1:1000; Jackson ImmunoResearch) in PBS. Following three washes with PBS, sections were exposed to the colorimetric diaminobenzidine reaction (DAB reagent, Dako) for 10 min and stopped with distilled water for 1 min. Finally, sections were dehydrated and mounted with DPX mounting medium (Sigma).

Images covering the entire slices were acquired at 10 × magnification for quantification with a high-throughput microscope (Nikon, Japan) with NIS elements software. Quantification of the mGluR1 immunoreactivity was performed using ImageJ software (National Institute of Health, USA). Images were converted into 8-bit grayscale, and an intensity threshold was applied to all images (threshold of 170 of 255). Regions-of-interest (ROIs) were manually drawn on each image, and the percentage of positive area after thresholding was assessed. Quantification was done in triplicate (3 slices) for each region, and the average was used for statistical analysis.

### Statistical Analysis

The Shapiro-Wilk test confirmed the normal distribution of the data. Longitudinal analysis of the PET data was performed using a linear mixed-model, given its robustness and the possibility to include subjects with missing observations. The linear mixed-model analysis was performed using *V*_T (IDIF)_ values as the dependent variable for each region separately, with genotype (WT and HET), time (6, 12, and 16 months), and the interaction between genotype and time (genotype*time) as fixed effects, and with subjects included as random effect, followed by multiple comparison correction for differences within and between genotypes performed using the Tukey-Kramer test. Regular two-way ANOVA with *post hoc* Bonferroni correction for multiple comparisons was applied to investigate differences between genotypes for *post-mortem* variables and reference region-based quantification. Linear mixed-model analysis was performed in JMP Pro 13 (SAS), while all the other analyses were performed with GraphPad Prism (v 6.0) statistical software. Data are represented as mean ± standard deviation (SD) unless specified otherwise; all tests were two-tailed, except for the voxel-based analyses. Statistical significance was set at *p* < 0.05.

## Results

### In Vivo mGluR1 Availability Was Higher in HD Mice

**Fig. 1:**
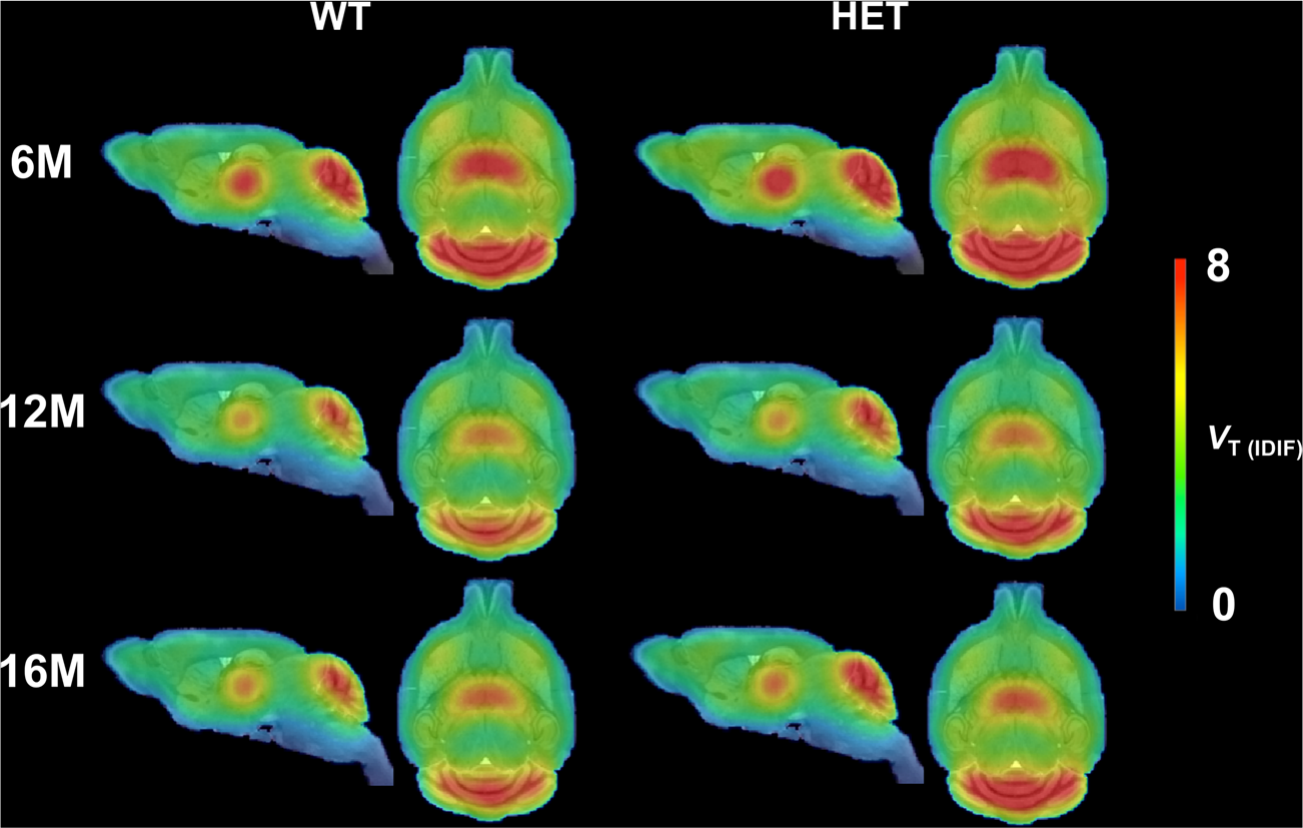
Average *V*_T (IDIF)_ (Logan) parametric maps of [^11^C]ITDM in WT and HET mice at each time point. Parametric maps are overlaid onto MRI mouse brain template for anatomical localization. WT: *n* = 19–21; HET: *n* = 18–19. WT = wild-type, HET = heterozygous, M = months

**Fig. 2:**
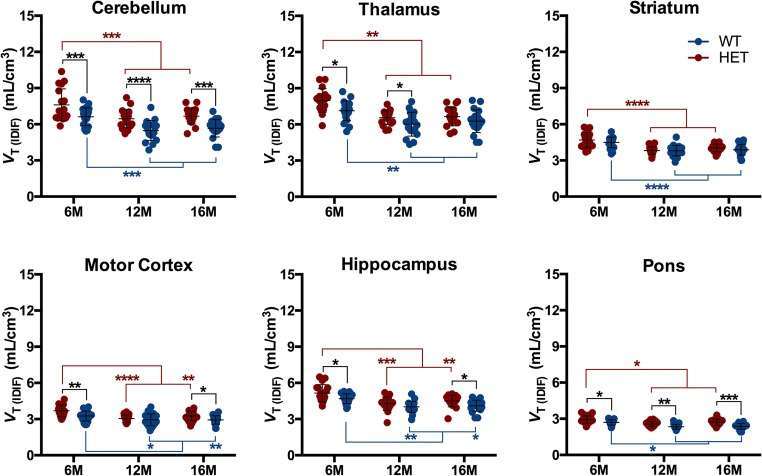
**Regional [**^**11**^**C]ITDM*V***_**T (IDIF)**_**quantification.** HET mice demonstrated higher [^11^C]ITDM *V*_T (IDIF)_ values compared to WT littermates. Both genotypes displayed an age-related temporal decline. Black stars indicate significance between genotypes, red stars within HET, and blue stars within WT. WT: *n* = 19–21; HET: *n* = 18–19. *p < 0.05, **p < 0.01, ***p < 0.001, ****p < 0.0001. WT = wild-type, HET = heterozygous, M = months

Mean parametric *V*_T (IDIF)_ Logan maps for WT and HET mice at each investigated time point are shown in Fig. 1. HET mice were characterized by elevated brain [^11^C]ITDM *V*_T (IDIF)_ values compared to WT littermates (Fig. 2). At 6 months of age, HET mice displayed higher [^11^C]ITDM *V*_T (IDIF)_ values than WT littermates in all investigated brain regions (e.g. cerebellum: WT = 6.61 ± 0.74 mL/cm^3^; HET = 7.61 ± 1.32 mL/cm^3^; + 15.1%, *p* < 0.0001), with the exception of striatum (WT = 4.49 ± 0.43 mL/cm^3^; HET = 4.69 ± 0.63 mL/cm^3^; + 4.4%, *p* > 0.05). At 12 months of age, the [^11^C]ITDM *V*_T (IDIF)_ values in HET mice were elevated compared to WT littermates only in cerebellum, thalamus, and pons: e.g., cerebellar *V*_T (IDIF)_ values were 5.48 ± 0.93 mL/cm^3^ for WT mice and 6.61 ± 0.74 mL/cm^3^ for HET littermates (+ 17.9%, *p* < 0.001). Finally, [^11^C]ITDM PET imaging at 16 months of age revealed that *V*_T (IDIF)_ values for HET mice were higher in cerebellum, motor cortex, hippocampus, and pons compared to WT littermates (e.g. cerebellum: *V*_T (IDIF)_ values of 5.66 ± 0.72 mL/cm^3^ for WT mice and 6.66 ± 0.65 mL/cm^3^ for HET littermates (+ 17.6%, *p* < 0.0001) (Fig. 2).

A significant temporal decline in [^11^C]ITDM *V*_T (IDIF)_ values was observed at 12 months of age in all investigated brain regions independently of the genotype. Accordingly, cerebellar *V*_T (IDIF)_ values between 6 and 12 months of age were reduced in both WT (− 17.2%, *p* < 0.001) and HET (− 15.1%, *p* < 0.001) mice. No further change was observed between 12 and 16 months of age (Fig. 2).

**Fig. 3:**
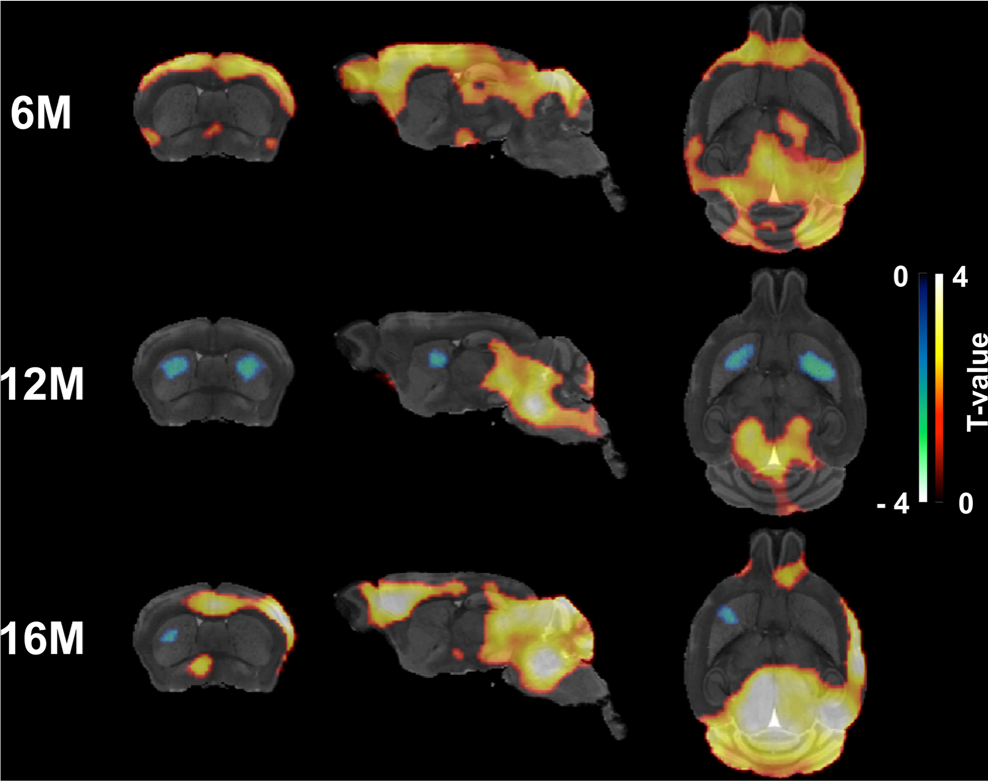
**Voxel-wise differences in [**^**11**^**C]ITDM binding between WT and HET mice.** Clusters of increased [^11^C]ITDM uptake in HET mice compared to WT littermates (HET > WT) are shown as hot scale, while clusters of reduced uptake in HET mice (WT > HET) as cold scale. WT: *n* = 19–21; HET: *n* = 18–19. M = months

Voxel-wise analysis of the parametric maps confirmed the extensive higher [^11^C]ITDM binding in HET mice. For instance, in line with the regional analysis, motor cortex displayed pronounced clusters of greater [^11^C]ITDM binding in HET mice at 6 months of age, which were not present at 12 months, but could be detected again at 16 months of age (Fig. 3). Intriguingly, voxel-wise analysis detected clusters of lower [^11^C]ITDM binding in striatum of HET mice at 12 and 16 months of age (Fig. 3), which could not be detected with the regional analysis.

### In Vitro mGluR1 Levels Were Greater in HD Mice

**Fig. 4:**
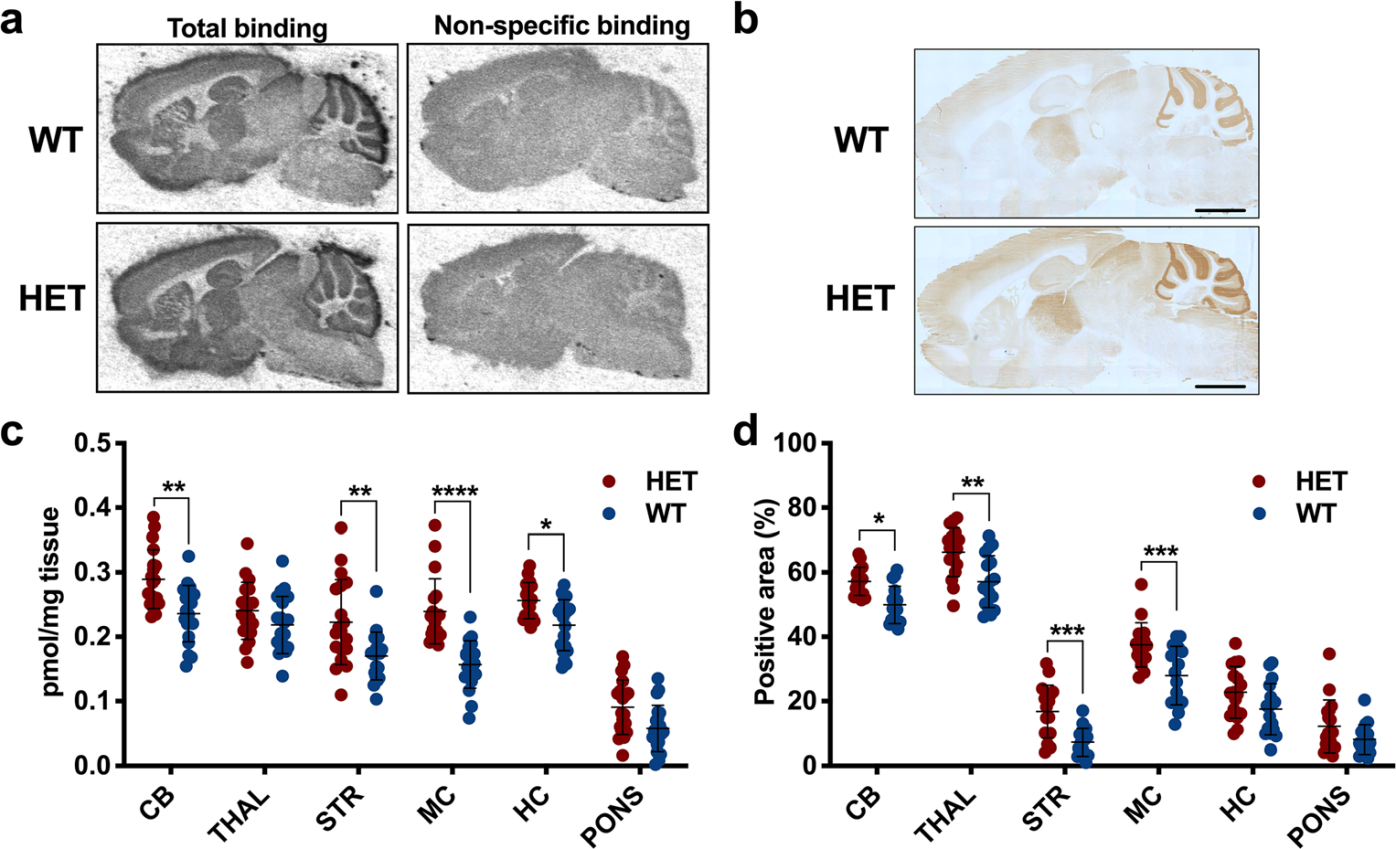
***Post-mortem*evaluation of mGluR1 at 16 months of age.** Representative **a** [^3^H]ITDM autoradiograms and **b** mGluR1 immunohistochemistry for WT and HET mice. **c** [^3^H]ITDM specific binding as well as **d** mGluR1 immunoreactivity were higher in HET mice compared to WT littermates. WT: *n* = 17–19; HET: *n* = 17–18. *p < 0.05, **p < 0.01, ***p < 0.001, ****p < 0.0001. Scale bar = 2 mm. WT = wild-type, HET = heterozygous, CB = cerebellum, THAL = thalamus, STR = striatum, MC = motor cortex, HC = hippocampus

*Post-mortem* quantification of mGluR1 was achieved using both [^3^H]ITDM autoradiography and mGluR1 immunohistochemistry in the same animals (WT, *n* = 19; HET, *n* = 18). Representative [^3^H]ITDM autoradiograms and mGluR1 immunoreactivity for WT and HET mice are shown in Fig. 4a and b, respectively. [^3^H]ITDM-specific binding was greater in cerebellum, striatum, motor cortex, and hippocampus of HET mice compared to WT littermates (Fig. 4c) (e.g. in cerebellum: 0.24 ± 0.04 pmol/mg for WT; 0.29 ± 0.04 pmol/mg for HET; + 21.7%, *p* = 0.010).

mGluR1 immunoreactivity, measured as percentage of positive area after thresholding, was significantly higher in cerebellum, thalamus, striatum, and motor cortex of HET mice compared to WT littermates (Fig. 4d) (e.g. in cerebellum: 49.9 ± 5.8% for WT; 57.2 ± 3.3% for HET; + 14.6%, *p* = 0.021).

### Reference Region-Based Analysis Biased the In Vivo mGluR1 Quantification

**Fig. 5:**
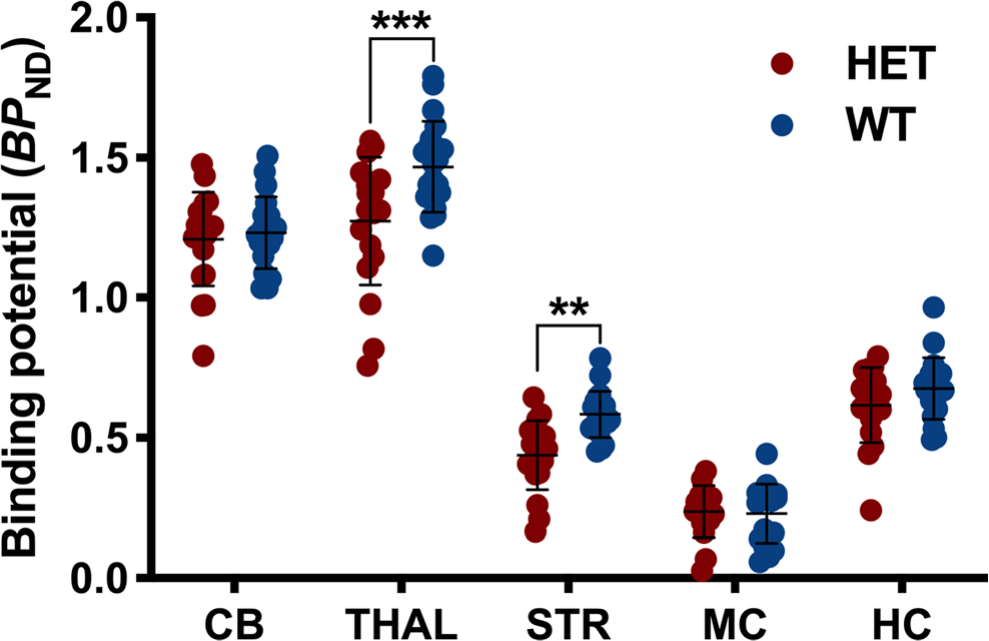
**Effect of reference region-based analysis on the in vivo [**^**11**^**C]ITDM quantification.** Relative quantification using pons as reference region resulted in contradictory results with higher binding potential in WT mice compared to HET littermates. Data at 16 months of age are reported. **p < 0.01, ***p < 0.001. WT = wild-type, HET = heterozygous, CB = cerebellum, THAL = thalamus, STR = striatum, MC = motor cortex, HC = hippocampus

In order to determine the effect of reference region-based quantification, we applied this approach to the time point in which we measured the largest genotypic difference (16 months of age). As shown in Fig. 5, relative quantification using pons as reference region resulted in contradictory results with HET mice displaying significantly lower values in thalamus (− 13.2%, *p* < 0.001) and striatum (− 24.9%, *p* < 0.01) compared to WT littermates.

## Discussion

To our knowledge, this is the first study to characterize temporal mGluR1 availability in HD. We used PET imaging with the radioligand [^11^C]ITDM to investigate changes in mGluR1 availability in HD mice during disease progression. Longitudinal PET imaging demonstrated an overall higher cerebral [^11^C]ITDM binding in HET mice compared to WT littermates at different stages of disease progression with the exception of striatum. The most pronounced difference was found in cerebellum, with higher mGluR1 levels ranging from 15.1% to 17.9% during HD progression. Of note, only a genotype-independent temporal decline in [^11^C]ITDM *V*_T (IDIF)_ was observed, indicating no disease-related effect. The observed decline is in agreement with previous investigation of the mGluR1 protein levels in the mouse cerebellum, in which a significant decline associated with age was demonstrated [34].

Only one report has previously examined in vivo mGluR1 availability in a mouse model of HD [29]. In contrast to our study, the authors reported a significant decrease in [^11^C]ITDM binding in the R6/2 model of HD; however, these results were obtained using a reference region-based (pons) kinetic modelling for quantification of tracer binding. Our group recently found that the pons is not a suitable reference region for [^11^C]ITDM as its binding in this region can be blocked and displaced both in vivo and in vitro [19]. Thus, application of reference tissue models can lead to incorrect results.

*Post-mortem* analysis showed a significant increase in [^3^H]ITDM-specific binding as well as greater mGluR1 immunoreactivity. This consistently supports the increase in mGluR1 levels of our in vivo [^11^C]ITDM PET imaging-based study. No *post-mortem* clinical investigation of mGluR1 has been performed yet, and only sparse evidence exists on the role of mGluR1 in HD at the preclinical level. Previous autoradiography studies investigating changes in the level of group I mGluRs were inconclusive, reporting only small trends, in both the R6/2 [35] and YAC128 [36] transgenic models of HD. Interestingly, we previously showed mGluR5 protein levels to be decreased in Q175 HD mice [17], while here, we demonstrated an increase in mGluR1 levels. Because of the opposing direction of mGluR1 and mGluR5 expression levels, previous studies on Group I mGluRs may have failed to detect differences due to the lack of subtype specificity.

One potential limitation of the current study was the lack of *post-mortem* validation at 6 and 12 months of age using satellite cohorts of animals. While such analysis could have provided a further indication of the temporal decline in mGluR1 levels measured in vivo, it was not performed as the aim of the *post-mortem* work was to confirm in vitro the changes measured in vivo in the same animals.

Interestingly, the voxel-wise PET analysis detected a selective decrease of in vivo striatal mGluR1 availability in HET mice primarily at 12 months with a small cluster still present at 16 months. In humans, mHTT accumulation originates in the striatum and progresses to other brain regions [37]. Since striatal medium spiny neurons are particularly vulnerable to mHTT-induced increased levels of intracellular Ca^2+^, the process of receptor desensitization and internalization [38] may be enhanced in order to compensate for the several glutamatergic inputs from the basal ganglia, thalamus, and cortex [39]. This would represent a protective process as previously postulated [10] and could explain the focal cluster of decreased in vivo mGluR1 availability observed in striatum with the voxel-based analysis. Conversely, *post-mortem* analyses showed increased striatal mGluR1 levels. This in vivo and in vitro restricted discrepancy may be a further indication that striatal mGluR1 is undergoing internalization in order to reduce the intracellular toxic levels of Ca^2+^.

A complex interplay exists between HTT and mGluR1/5. HTT and HTT-associated protein 1 have been shown to regulate the levels of intracellular Ca^2+^ by interacting with IP_3_R1 [40]. On one hand, mHTT enhances this signalling pathway, resulting in toxic levels of intracellular Ca^2+^ and intensified activation of protein kinase C (PKC), which desensitize the mGluR1/5 receptors [40, 41]. On the other hand, PKC also increases other downstream pathways including extracellular regulated kinase (ERK) and protein kinase B (also known as Akt) [42], which may promote cell survival and facilitate clearance of mHTT species. In addition, mHTT impairs the functionality of the Rab8/optineurin complex [43]. Since Rab8 modulates the trafficking and signalling of mGluR1 [44], the observed mGluR1 increase may represent a combination of an adaptive response to promote cell survival and impairment in regulation of mGluR1 surface localization. In this perspective, mGluR1 has the potential to represent a therapeutic target analogously to mGluR5 as recently reported [12, 45, 46]. Nonetheless, due to the intricate mGluR1/5 downstream signalling pathways, it is challenging to interpret the pathological effect of these changes. Further research is needed to gain a better understanding of the potential therapeutic significance of the measured mGluR1 changes during the course of disease in this HD model and to validate these findings clinically.

## Conclusion

We report quantification of in vivo mGluR1 availability using [^11^C]ITDM PET imaging during disease course in the Q175DN mouse model of HD. These findings demonstrated higher [^11^C]ITDM binding in extra-striatal brain regions during disease progression in HD mice, further corroborating HD as a complex whole brain disorder.

## Electronic supplementary material

ESM 1 (DOCX 154 kb)
