# Supplementary material for: Elevated Type 1 Metabotropic Glutamate Receptor Availability in a Mouse Model of Huntington’s Disease: a Longitudinal PET Study
Source: Mol Neurobiol. 2020 Jan 8;57(4):2038–47. doi: 10.1007/s12035-019-01866-5 (PMC7118044; doi:10.1007/s12035-019-01866-5)
Supplement: Supplementary file 1 — (DOCX 154 kb) [file 12035_2019_1866_MOESM1_ESM.docx]

**Elevated type 1 metabotropic glutamate receptor availability in a mouse model of Huntington’s Disease:
a longitudinal PET study**

Daniele Bertoglio^1^, Jeroen Verhaeghe^1^, Špela Korat^1,2^, Alan Miranda^1^, Klaudia Cybulska^1,2^, Leonie Wyffels^1,2^, Sigrid Stroobants^1,2^, Ladislav Mrzljak^3^, Celia Dominguez^3^, Mette Skinbjerg^3^, Longbin Liu^3^, Ignacio Munoz-Sanjuan^3^, Steven Staelens^1^

^1^Molecular Imaging Center Antwerp (MICA), University of Antwerp, Wilrijk, Belgium

^2^Department of Nuclear Medicine, Antwerp University Hospital, Edegem, Belgium

^3^CHDI Management/CHDI Foundation, Los Angeles, California, USA

**Corresponding author:**

Prof. Steven Staelens

Email: [steven.staelens@uantwerpen.be](mailto:steven.staelens@uantwerpen.be)

**Supplementary Table**

**Supplementary Table 1.** Imaging scan parameters for both WT and HET Q175DN animals at each time point.

| **Age (months)** | **Genotype** | **number of animals** | **molar radioactivity (GBq/μmol)** | **injected dose (MBq)** | **injected mass (μg/kg)** | **body weight (g)** |
| --- | --- | --- | --- | --- | --- | --- |
|  |  |  |  |  |  |  |
| **6** | **WT** | 21 | 90.0 ± 25.4 | 4.9 ± 0.7 | 1.11 ± 0.37 | 32.2 ± 2.9 |
|  | **HET** | 19 | 99.6 ± 34.9 | 4.9 ± 0.7 | 1.11 ± 0.45 | 30.0 ± 1.7 |
| **12** | **WT** | 20 | 79.0 ± 28.0 | 6.3 ± 2.0 | 1.46 ± 0.25 | 36.7 ± 5.1 |
|  | **HET** | 18 | 81.7 ± 26.7 | 4.8 ± 1.4 | 1.40 ± 0.23 | 28.0 ± 1.8 |
| **16** | **WT** | 19 | 91.3 ± 28.8 | 6.0 ± 1.7 | 1.25 ± 0.23 | 36.8 ± 5.4 |
|  | **HET** | 18 | 87.3 ± 27.2 | 4.4 ± 1.4 | 1.39 ± 0.25 | 25.6 ± 2.5 |

WT = wild-type, HET = heterozygous. Values are expressed as mean ± SD.

**Supplementary Figures**

**Supplementary Fig. 1 Average image-derived input function (IDIF) time-activity curves (TACs).** Average IDIF TACs of WT and HET animals did not show any significant difference at **a** 6 months, **b** 12 months, or **c** 16 months. WT: *n* = 19-21; HET: *n* = 18-19. Mean ± s.e.m.

**Supplementary Fig. 2 *In vivo* metabolism of [^11^C]ITDM in WT and HET animals.** Population-based curve of intact [^11^C]ITDM measured in plasma of WT and HET Q175DN mice at 6 months of age. *n* = 3 per genotype at each time point.
